# Tri-methylation of H3K79 is decreased in TGF-β1-induced epithelial-to-mesenchymal transition in lung cancer

**DOI:** 10.1186/s13148-017-0380-0

**Published:** 2017-08-08

**Authors:** Emilie Evanno, Julie Godet, Nathalie Piccirilli, Joëlle Guilhot, Serge Milin, Jean Marc Gombert, Benoit Fouchaq, Joëlle Roche

**Affiliations:** 1 Eurofins Cerep SA, Le Bois l’Evêque, F-86600 Celle L’Evescault, France; 2 Université de Poitiers, Laboratoire LNEC, F-86022 Poitiers, France; 3 CHU de Poitiers, Service d’Anatomie et de Cytologie Pathologiques, F-86021 Poitiers, France; 4 INSERM U1082, CHU de Poitiers, F-86021 Poitiers, France; 5 INSERM CIC 0802, CHU de Poitiers, F-86021 Poitiers, France; 6 Service Immunologie, CHU de Poitiers, F-86021 Poitiers, France; 7 Laboratoire Ecologie et Biologie des Interactions (EBI), Université de Poitiers, UMR-CNRS 7267, F-86073 Poitiers, France

**Keywords:** Lung cancer, NSCLC, EMT, H3K79me3, DOT1L, PD-L1, SEMA3C, NRP2, Epigenetic treatment

## Abstract

**Background:**

The epithelial-to-mesenchymal transition (EMT) enables epithelial cancer cells to acquire mesenchymal features and contributes to metastasis and resistance to treatment. This process involves epigenetic reprogramming for gene expression. We explored global histone modifications during TGF-β1-induced EMT in two non-small cell lung cancer (NSCLC) cell lines and tested different epigenetic treatment to modulate or partially reverse EMT.

**Results:**

Loss of classical epithelial markers and gain of mesenchymal markers were verified in A549 and H358 cell lines during TGF-β1-induced EMT. In addition, we noticed increased expression of the axonal guidance protein semaphorin 3C (SEMA3C) and PD-L1 (programmed death-ligand 1) involved in the inhibition of the immune system, suggesting that both SEMA3C and PD-L1 could be the new markers of TGF-β1-induced EMT. H3K79me3 and H2BK120me1 were decreased in A549 and H358 cell lines after a 48-h TGF-β1 treatment, as well as H2BK120ac in A549 cells. However, decreased H3K79me3 was not associated with expression of the histone methyltransferase DOT1L. Furthermore, H3K79me3 was decreased in tumors compared in normal tissues and not associated with cell proliferation. Associations of histone deacetylase inhibitor (SAHA) with DOT1L inhibitors (EPZ5676 or SGC0946) or BET bromodomain inhibitor (PFI-1) were efficient to partially reverse TGF-β1 effects by decreasing expression of PD-L1, SEMA3C, and its receptor neuropilin-2 (NRP2) and by increasing epithelial markers such as E-cadherin.

**Conclusion:**

Histone methylation was modified during EMT, and combination of epigenetic compounds with conventional or targeted chemotherapy might contribute to reduce metastasis and to enhance clinical responses.

**Electronic supplementary material:**

The online version of this article (doi:10.1186/s13148-017-0380-0) contains supplementary material, which is available to authorized users.

## Background

The epithelial-to-mesenchymal transition (EMT) is a highly dynamic and reversible mechanism by which epithelial cells can convert into a mesenchymal phenotype, allowing a loss of cellular adhesion, cellular polarity, and an improvement in migratory and invasive properties. This process occurs during embryonic development, wound healing, and metastatic expansion [1–3]. It also plays a major role in resistance to cancer treatment [4, 5]. A major inducer of EMT is TGF-β1, along with cytokines and growth factors secreted by the tumor microenvironment. Switch in gene expression during EMT is characterized by repression of epithelial genes and induction of mesenchymal genes.

The induction of EMT is associated with reprogramming of the epigenome characterized by chromatin remodeling, changes in DNA methylation, post-translational histone modifications or insertion of histone variants, and modifications of non-coding RNA expression [2, 6, 7]. The basic unit of chromatin, the nucleosome, is formed by 180–200 bp of DNA wrapped around a histone protein complex, composed by an octamer of two copies of each histone H2A, H2B, H3, and H4, and fixed by histone H1 [8]. Each histone can be affected by post-translational modifications (PTMs), including acetylation, methylation, phosphorylation, ubiquitination or sumoylation, that define a complex “histone code.” PTMs are regulated by histone-modifying enzymes which add or erase these modifications. They are recognized and read by protein partners to control the accessibility of the transcriptional machinery to nearby genes [9]. All these processes can be considered as epigenetic-based therapeutic strategy to treat cancer [10].

Lung carcinomas cause one fifth of cancer deaths worldwide [11]. Large-scale genomic studies have characterized frequent modifications affecting epigenetic mechanisms [12–14]. Recurrent mutations of epigenetic modifying genes affect the SWI/SNF chromatin remodeling components (ARID1a and BRG1) and the H3K36 methyltransferase SETD2 in about 20% of lung adenocarcinomas [15, 16]. The H3K79 methyltransferase DOT1L mutations are less frequent and are described in 3% of lung adenocarcinomas [17]. In squamous lung cancers, mutations of the H3K4 methyltransferase MLL2 are described in 20% [18]. Abnormal expression of epigenetic enzymes includes overexpression of EZH2 (enhancer of zeste homolog 2), the catalytic subunit of the PRC2 repressive complex that methylates H3K27, associated with tumor progression and poor prognosis in lung cancer [19–22]. Abnormal epigenetic marks are noticed in lung cancer, mainly DNA methylation, histone acetylation, and methylation for the most studied marks [14]. DNA methylation and miRNAs have emerged as potential biomarkers in body fluids for lung cancer [13, 23].

During EMT, the transcription factors SNAIL and ZEB1 recruit several epigenetic players, including histone methyltransferases, the lysine demethylase LSD1, HATs (histone acetyltransferases) and HDACs (histone deacetylases), sirtuins, and BRG1 [2]. We reported a global decrease in H3K27 acetylation in a ZEB1-induced EMT lung cancer cell model and suggested that ZEB1 would recruit EZH2 [24]. EMT is also associated with repression of the miR-200 family. However, EMT-related epigenetic reprogramming is still poorly understood in lung tumors.

In the present study, we focused on epigenetic modifications during TGF-β1-induced EMT in non-small cell lung cancer (NSCLC) cells and tested different epigenetic treatment to modulate or partially reverse EMT.

## Methods

### Cell lines, inhibitors, and antibodies

NSCLC cell lines, A549, and NCI-H358 (hereafter H358) were obtained from ATCC in 2014. The cells were grown in RPMI-1640 medium with 10% fetal bovine serum (FBS) and antibiotics-antimycotics (#15240-062, Invitrogen, Carlsbad, CA, USA), at 37 °C and 5% CO_2,_ and controlled every month for mycoplasma contamination. Recombinant human TGF-β1 was from R&D Systems (Minneapolis, MN, USA). The inhibitors EPZ5676, SGC0946, PFI-1, and SAHA were purchased from Cayman (Ann Arbor, Michigan, USA). The antibodies are listed in Additional file 1.

### RNA extraction and quantification by real-time quantitative PCR

Total RNA was extracted using the RNeasy Mini kit (Qiagen, Hilden, Germany) following the manufacturer’s instructions. RNA quality was controlled by electrophoresis on 0.8% agarose gel. Five hundred nanograms of total RNA was reverse-transcribed using the iScript Reverse Transcription Kit (Biorad, Hercules, CA, USA). Real-time quantitative PCR (qPCR) was performed using SsoAdvanced Universal SYBR Green Supermix (Bio-Rad) on a CFX384 system (Bio-Rad). Data were analyzed using the Bio-Rad CFX Manager software and normalized to GAPDH messenger RNA (mRNA) level using the 2^−ΔCq^ method. The primer sequences are listed in Additional file 2.

### Protein detection by immunoblotting

Cells were directly lysed in Laemmli loading buffer and sonicated. Whole-cell extracts were separated on SDS-PAGE gels (4–15% acrylamide gradient, #4568084, Bio-Rad) and transferred to 0.2 μm nitrocellulose membrane (#1704159, Bio-Rad) with the Trans-Blot Turbo Transfer System (Bio-Rad) for 7 min. The membranes were blocked with 5% BSA in PBS 0.05% Tween (PBS-T) for 90 min. They were then incubated with primary antibodies at the indicated dilutions (Additional file 1) in 5% BSA in PBS-T, at 4 °C overnight. Removal of excess primary antibodies was carried out by washing the membranes in PBS-T (3 × 5 min each). Secondary antibodies were incubated with the membranes in 5% BSA-PBS-T for 1 h at room temperature. The membranes were washed in PBS-T (3 × 5 min each) before exposition to Clarity Western ECL substrate (#1705060, Bio-Rad). Blot images were acquired with the ChemiDoc MP System (Bio-Rad) and quantified with the Image Lab software (Bio-Rad). Protein normalization was carried out with anti-actin or anti-total H3 histone staining.

### Immunofluorescence

Cells grown on 8-well Ibidi plate (Martinsried, Germany) were fixed for 15 min with 4% paraformaldehyde. After rinsing with PBS (3 × 5 min), the cells were permeabilized for 20 min with 0.2% Triton X-100 in PBS. After rinsing (3 × 5 min with PBS), blocking was performed with 3% normal chicken serum (#ab7477, Abcam, Cambrigde, UK) in PBS during 1 h. Primary and secondary antibodies were sequentially applied for 60 min at room temperature in 3% serum in PBS at the indicated dilutions (Additional file 1). DRAQ5 at 1:250 dilution (Thermo Scientific, Rockford, IL, USA) was applied with the secondary antibody for DNA staining. Stained slides were rinsed in PBS and mounted in Dako fluorescent media (Santa Clara, CA, USA). Images were captured with a confocal microscope (Olympus FluoView FV1000) at ×60 immersion oil objective.

### Flow cytometry

5 × 10^5^ cells were requested per condition and were diluted at 5 × 10^6^ cells/ml in PBS 2% FBS. Near-IR staining (#L10119, Thermo Fisher, Waltham, MA, USA) was performed during 20 min at 4 °C to detect dead cells. The cells were washed once in PBS 2% FBS and stained for 1 h at 4 °C with PD-L1 antibody or BB515 isotype control at the indicated dilutions (Additional file 1). BB515 IgG1 isotype control is used as a negative control and binds specifically to KLH antigen which is not expressed in human cells. Flow cytometry analyses were conducted on a BD FACSVerse flow cytometer (BD Biosciences, San Jose, CA, USA). Data were analyzed using the FlowJo software (Tree Star, Ashland, OR, USA).

### Cancer tissue microarray (TMA) and immunohistochemistry

Three commercial formalin-fixed, paraffin-embedded TMA slides were obtained from USBiomax (Rockville, MD, USA). LC121a is a lung cancer TMA (120 samples), MC6163 a multi-tissue TMA (616 samples) that includes normal and cancer tissues, and BCN962 a combined multiple normal and cancer tissue microarray (96 samples), with 17 types of common organs. Both TMAs LC121a and MC613 contain 110 and 48 lung cancer tissues, respectively, with evaluation of TNM disease stages. TMA slides were deparaffinized by incubations (2 × 3 min) in Histosol solution (National Diagnostics, Inc., Charlotte, NC, USA) and rehydrated by sequential immersions in 100% ethanol (2 × 1 min) and 70% ethanol (1 min). After washing 2 min in water, the slides were incubated for 2 min in a pressure cooker with the antigen unmasking solution (10 mM citrate buffer, pH = 6) and left for cooling for additional 20 min in the de-pressurized cooker. After washing 5 min in TBS-Tween (Dako Wash Buffer #S3006, Glostrup, Denmark), endogenous peroxidases were inhibited with 3% H_2_O_2_-PBS during 5 min at room temperature. Following washing in TBS-Tween (1 × 5 min), the tissues were permeabilized with 0.2% Triton in PBS for 20 min. Blocking was performed with 3% BSA-PBS during 20 min. Both TMAs LC121a and MC613 were incubated for 1 h at room temperature in a wet chamber with rabbit anti-H3K79me3 primary antibody (1:200; #pAb-068-050, Diagenode, Denville, NJ, USA,). For BCN962 slides, rabbit anti-H3K79me3 primary antibody was incubated for 1 h at room temperature or with mouse anti-Ki67 primary antibody (1:100; #M7240, Dako) in Emerald diluent (#936b-08, Cell Marque, Rocklin, CA, USA) overnight at 4 °C. Following washing in TBS-Tween (1 × 5 min), the slides were incubated for 30 min with the HRP-labeled polymer conjugated with secondary antibodies (#K4065, EnVision® + Dual Link System-HRP kit, Dako). Slides were washed 5 min in TBS-Tween and further incubated in 3,3-diaminobenzidine (DAB) for 5 min. After washing in H_2_O, tissues were incubated in hematoxylin for 2 min. TMA slides were dehydrated by sequential immersions in 70% ethanol (1 min) and 100% ethanol (2 × 1 min) and finally mounted using mount medium (#10046430011, Leica, Wetzlar, Germany). For TMAs LC121a and MC613, each sample was scored by multiplying the percentage of positive (0 to 100%) cells in the tumor compartment by the average level of staining intensity (0 to 3). For TMA BCN962, Ki67 expression was determined by the percentage of positive (0 to 100%) epithelial cells in the tumor compartment and H3K79me3 expression was analyzed by the level of staining (0 to 3) for epithelial cells only.

### Statistical analysis

Data were summarized by median and range for quantitative variables, percentage, and confidence intervals when appropriate for qualitative variables. Percentage of positive cells staining intensity and expression score distributions in tumor, stroma, and normal tissues were compared using Wilcoxon rank-sum test and the Kruskal-Wallis test. Relationship between co-staining status and qualitative parameters (histology, nodes) was analyzed by Fisher’s exact test or chi-square as appropriate. Correlations between score expression and other quantitative parameters were determined with the Spearman rank correlation method. Potential relationships with baseline characteristics were also explored with the use of non-parametric test, as appropriate.

## Results

### TGF-β1 exposure induces EMT in NSCLC cell lines

**Fig. 1:**
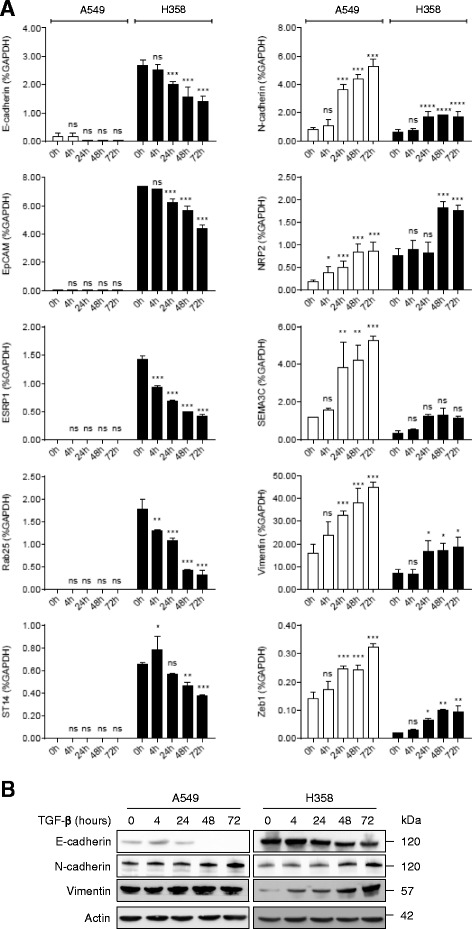
TGF-β1 induces EMT in NSCLC cell lines. Expression of epithelial and mesenchymal markers was determined by mRNA and protein analysis of A549 (white bars) and H358 (black bars) cells cultured with TGF-β1 (10 ng/ml) for the indicated time (h). **a** mRNA expression was measured by RT-qPCR and normalized to GAPDH mRNA level. The graph corresponds to the mean ± SD of two independent experiments with PCR in duplicate. *p < 0.5, **p < 0.01, ***p < 0.001 by a two-way ANOVA test. **b** Protein expression was determined by immunoblotting of the A549 and H358 total cell lysates. Actin was used as a loading control. The apparent molecular weights (kDa) are indicated on the *right* of the panel

A549 and H358 NSCLC cell lines were selected for EMT induction by TGF-β1 because of their previous characterization in a ZEB1-induced EMT model [25]. We first verified target gene expression over time during a 72-h period to validate our cellular models. As expected, we could verify the more epithelial status of H358 cells compared to that of A549 cells. Upon TGF-β1 treatment, the progressive loss of the classical epithelial marker like E-cadherin was noticed in addition to the gain of the mesenchymal markers N-cadherin and vimentin (Fig. 1a, b). Expression of other epithelial markers ST14, ESPR1, EpCAM, and Rab25 were also decreased like in the ZEB1-induced EMT model [24, 25]. ZEB1, one of the transcription factors involved in EMT induction, was increased as expected (Fig. 1a). Since EMT is often partial and NSCLC cell lines display intermediate/hybrid states with mixed epithelial and mesenchymal characteristics [26], we checked the expression of additional genes coding for the guidance proteins, semaphorin 3C (SEMA3C) and semaphorin 3F (SEMA3F), and one of their common receptors, neuropilin-2 (NRP2), because of their involvement in lung cancer progression and EMT [27, 28]. In both cell lines, SEMA3F expression did not change (result not shown), but SEMA3C and NRP2 were increased (Fig. 1a) suggesting a more aggressive phenotype upon TGF-β1 treatment. From these collected data, a 48-h TGF-β1 treatment was chosen for EMT induction for further analysis.

### TGF-β1-induced EMT increases PD-L1 expression in H358 cells

For a better characterization of TGF-β1-induced EMT, we analyzed the programmed death-ligand 1 (PD-L1) expression in A549 and H358 cells. PD-L1 plays a critical role by associating programmed death 1 receptor (PD-1) on tumor-infiltrating T cells thus inhibiting the immune response. Indeed, in 1070 surgically resected NSCLC specimens, PD-L1 was expressed in 44% of them and strong PD-L1 staining correlated with poor prognostic [29]. Interestingly, EMT is associated with an inflammatory tumor microenvironment in lung adenocarcinoma [30].

**Fig. 2:**
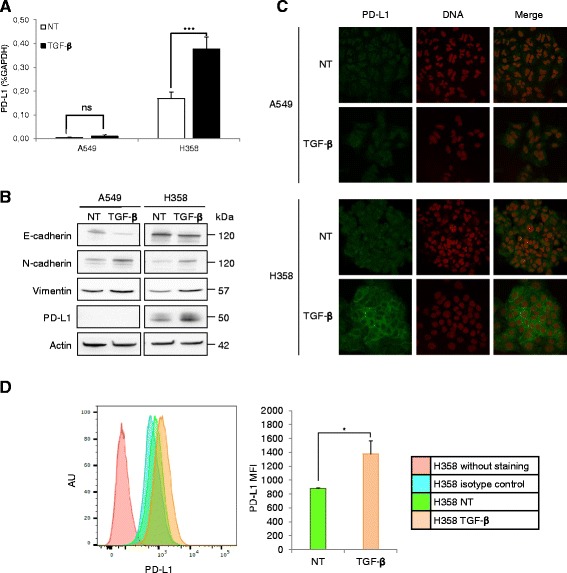
TGF-β1-induced EMT increases PD-L1 expression in H358 cells. Cells were treated with TGF-β1 (10 ng/ml) for 48 h. **a** PD-L1 mRNA expression was measured by RT-qPCR and normalized to GAPDH mRNA. The graph corresponds to the mean ± SD of three independent experiments. ***p < 0.001 by Student’s *t* test. **b** PD-L1 expression was determined by immunoblotting of cell lysates. Actin was used as a loading control. The apparent molecular weights (kDa) are indicated on the right of the panel. **c** PD-L1 (green) was detected by immunofluorescence and analyzed by confocal microscopy. DNA (red) was stained with DRAQ5. **d** Representative flow cytometry experiment of PD-L1 staining in H358 cells. Histograms represent the mean ± SD of PD-L1 MFI (mean fluorescence intensity) of three independent experiments (right panel). *p = 0.0391 by Student’s *t* test. *NT* no TGF-β1 treatment

Untreated H358 cells were found positive for PD-L1, whereas PD-L1 was not detectable in A549 cells previously described as a negative control in several studies [30, 31] (Fig. 2a, b). After a 48-h TGF-β1 treatment, PD-L1 was still not detected in A549 cells but was increased at the mRNA and protein levels in H358 cells (Fig. 2a, b). Immunofluorescence confirmed increased membranous PD-L1 staining (Fig. 2c), and a 60% increase in intensity was estimated by flow cytometry (Fig. 2d). Therefore, H358 cells would become more aggressive upon TGF-β1 treatment.

### H3K79 methylation is decreased in TGF-β1-induced EMT

**Fig. 3:**
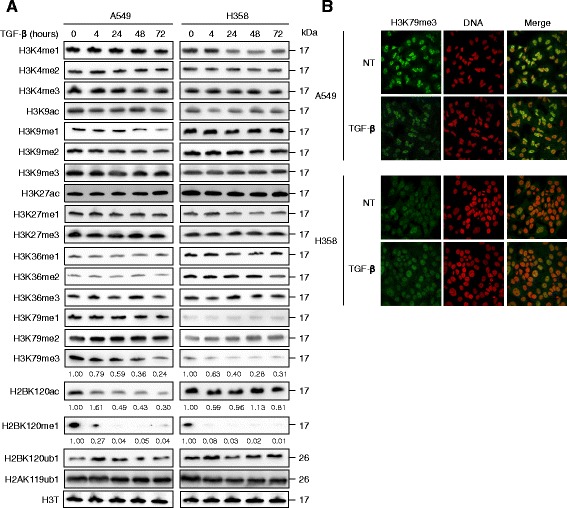
TGF-β1-induced EMT promotes histone post-translational modifications. A549 and H358 cells were treated with TGF-β1 (10 ng/ml) for the indicated time. **a** Histone marks were detected by immunoblotting from total cell lysates. H3 total (H3T) was used as a loading control. The apparent molecular weights (kDa) are indicated on the right of the panel. The intensity of the protein bands was quantified after normalization to H3T and compared to control cells (0 h) standardized to one. **b** H3K79me3 (green) was detected by confocal microscopy. DNA (red) was stained with DRAQ5

We investigated global histone modifications during EMT induction in both A549 and H358 cell lines. Of interest, untreated A549 and H358 cells present differences for some of the tested histone marks, such as H3K9me1, -me2, H3K36me1, -me2, H3K79me1, -me2, -me3, and H2BK120ac. Whereas most global histone marks did not change, up to 72-h TGF-β1 treatment, some of them including H3K79me3 and H2BK120me1, decreased in both cell lines (Fig. 3a). Of note, A549 cells have a higher basal level of H3K79me1, -me2, and -me3 than H358 cells. Immunocytochemistry confirmed H3K79me3 decrease in A549 cells after a 48-h treatment, but variation of H3K79me3 in H358 cells was difficult to estimate because of the low basal level (Fig. 3b). The different methylation states of H3K79 are generated in a distributive manner by the histone methyltransferase DOT1L [32], and the di-methylation of H3K79 is activated by ubiquitination of H2BK120 [33], suggesting that H2BK120ub1 may associate with DOT1L to promote H3K79 methylation [34]. However, H2BK120ub1 level was not modified in A549 neither in H358 cells during EMT induction, but H2BK120me1 was decreased (Fig. 3a). H2BK120ac was also decreased in A549 cells. This result suggests that H2BK120ub1 global level may not be directly involved in the reduction of H3K79me3 but that other modifications like acetylation and methylation of H2BK120 could be.

**Fig. 4:**
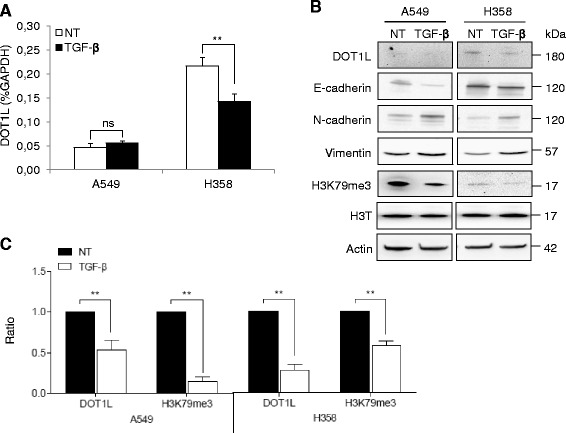
TGF-β1-induced EMT represses DOT1L in H358 cells. Cells were treated with TGF-β1 (10 ng/ml) for 48 h. **a** DOT1L mRNA expression was measured by RT-qPCR and normalized to GAPDH mRNA. The graph corresponds to the mean ± SD of three independent experiments with PCR in duplicate. **p = 0.0056 by Student’s *t* test. **b** DOT1L protein was analyzed by immunoblotting of A549 and H358 total cell lysates. Actin was used as a loading control. The apparent molecular weights (kDa) are indicated on the right of the panel. This blot is representative of four independent experiments. **c** The graph corresponds to the quantification of the intensity of the protein bands for A549 and H358 cell lines with (white bars) or without TGF-β1 (black bars) treatment after normalization to actin for DOT1L and total histone H3 for H3K79me3. Value 1 corresponds to untreated cells. Statistical analysis was performed for four independent experiments with the Student’s *t* test (** corresponds to *p* < 0.01)

Next, we investigated DOT1L expression. After a 48-h treatment, DOT1L expression was decreased at the mRNA and protein levels in both cell lines (Fig. 4a, b). Of note and surprisingly, H358 cells express more DOT1L than A549 cells but H3K79me3 is less, suggesting a complex mechanism for H3K79 methylation.

### Partial inhibition of EMT induction by epigenetic compounds

**Fig. 5:**
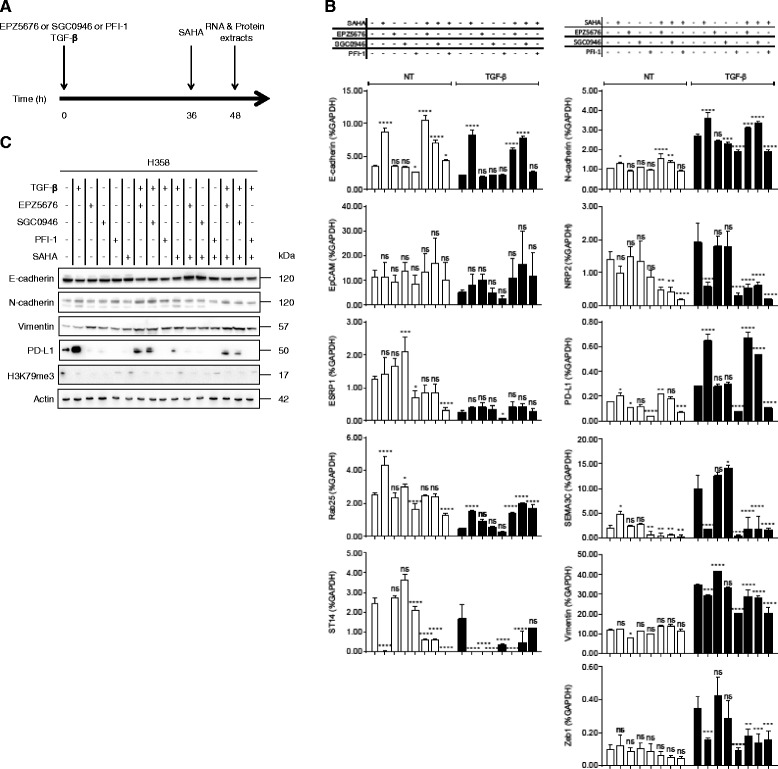
Partial reversion of TGF-β1-induced EMT by epigenetic inhibitors in H358 cells. **a** Protocol: Cells were treated simultaneously with TGF-β1 (10 ng/ml), EPZ5676 (1 μM), SGC0946 (5 μM), or PFI-1 (5 μM) for 48 h and with SAHA (5 μM) for the last 12 h. **b** mRNA expression was measured by RT-qPCR and normalized to GAPDH mRNA for control cells (white bars) and TGF-β1 treated cells (black bars). The graph corresponds to the mean ± SD of two independent experiments with PCR in duplicate. *p < 0.5, **p < 0.01, ***p < 0.001, ****p < 0.0001 by a two-way ANOVA test. **c** Proteins were analyzed by immunoblotting of total cell lysates. Actin was used as a loading control. The apparent molecular weights (kDa) are indicated on the right of the panel

Epigenetic compounds were tested to reverse EMT in untreated cells and to inhibit EMT induced with TGF-β1 (Fig. 5). Results are summarized in Table 1. First, in order to evaluate the involvement of H3K79me3 in TGF-β1-induced EMT, A549 and H358 cells were treated with DOT1L inhibitors, EPZ5676 and SGC0946, simultaneously with TGF-β1 (Fig. 5a). The efficacy of these compounds was verified by H3K79me3 decrease (Fig. 5c). In absence of TGF-β1, these compounds did not modify expression of EMT-related genes except for an increase of ESRP1 with SGC0946 (Fig. 5b). In combination with TGF-β1, both DOT1L inhibitors did not reverse expression induced by TGF-β1 for the selected genes except for PD-L1 that was reduced at the protein level in H358 cells (Fig. 5c).

**Table 1:** Epigenetic treatments in H358 cells

H358 cells		Epithelial genes					Mesenchymal genes					
		E-cadh	EpCAM	ESRP1	Rab25	ST14	N-cadh	NRP2	PD-L1	SEMA3C	Vim	ZEB1
Without TGF-β1	SAHA	*2.51*			*1.70*	0.0053	*1.25*		*1.33*	*2.35*		
	EPZ5676								0.69		0.675	
	SGC0946			*1.66*	*1.19*							
	PFI-1	0.73		0.56	0.65	0.85			0.26	0.28		
	SAHA + EPZ5676	*3.01*				0.24	*1.52*	0.33	*1.40*	0.20		
	SAHA + SGC0946	*2.02*				0.24	*1.33*	0.29		0.31		
	SAHA + PFI-1	*1.25*		0.25	0.51	0.0038		0.13	0.45	0.14		
With TGF-β1	SAHA	*3.90*			*3.36*	0.0002	*1.34*	0.30	*2.32*	0.17	0.84	0.45
	EPZ5676					0.0004					*1.21*	
	SGC0946					0.0016	0.86			1.43		
	PFI-1			0.18		0.21	0.71	0.15	0.27	0.03	0.58	0.26
	SAHA + EPZ5676	*2.83*			*3.00*	0.0001	*1.15*	0.27	*2.40*	0.17	0.82	0.51
	SAHA + SGC0946	*3.74*			*4.42*	0.27	*1.25*	0.32	*1.91*	0.18	0.82	0.39
	SAHA + PFI-1				*3.71*		0.71	0.10	0.37	0.15	0.59	0.45

Statistical significant results from Fig. 5 are summarized for variation of gene expression upon different treatments in absence or presence of TGF-β1, with the ratio of RT-qPCR values of treated cells/corresponding untreated control cells. Values above 1 (italics) indicate increased expression, and values under 1 indicate decreased expression

**Fig. 6:**
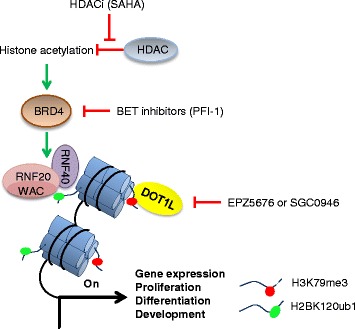
H3K79 methylation pathway and potential therapeutic targets. Histone acetylation is recognized and bound by BET bromodomain proteins (e.g., BRD4) which induced the protein complex RNF20/RNF40/WAC activation and H2BK120 ubiquitination. DOT1L which activity is stimulated by H2BK120ub1 further methylates H3K79. BRD4 and DOT1L can be inhibited by PFI-1 and EPZ5676/SGC0946, respectively

Histone acetylation is involved in DOT1L activation through H2B ubiquitination [35] (Fig. 6). Combined treatments with inhibitors of BET family proteins that are readers for acetylation, and inhibitors of histone deacetylases, induced anti-cancer effects in mouse models [36, 37]. For these reasons, we examined the impact of PFI-1 (a BRD2 and BRD4 inhibitor) and SAHA (a HDAC inhibitor). On control H358 cells, SAHA increased E-cadherin, Rab25, and decreased ST14 expression suggesting a partial gain of an epithelial phenotype (Fig. 5b, c). When added to TGF-β1, SAHA increased E-cadherin, Rab25, N-cadherin, and PD-L1 expression but decreased NRP2, SEMA3C, ST14, and ZEB1 expression compared to the TGF-β1 control.

PFI-1 treatment in H358 control cells, by itself, did not induce gross modifications but interestingly decreased PD-L1 and SEMA3C expression. When combined with TGF-β1, the major effect of PFI-1 was to decrease NRP2, PD-L1, SEMA3C, vimentin, and ZEB1 expression (ratio treated/untreated, 0.15, 0.27, 0.03, 0.58, and 0.26, respectively) to levels similar to control cells. However, expression of epithelial genes such as ESRP1 and ST14 was decreased. Next, DOT1L inhibitors or PFI-1 were combined with SAHA in presence or absence of TGF-β1 treatment. Interestingly, all tested combinations were able to reduce NRP2, SEMA3C, and ZEB1 in presence of TGF-β1. The combination between DOT1L inhibitors and SAHA was sufficient to increase E-cadherin expression in H358 cells (Fig. 5b). The addition of SAHA to PFI-1 improved the response compared to PFI-1 alone to increase E-cadherin expression and to decrease NRP2 expression in control cells. This combination did not improve the PFI-1 effect in TGF-β1-treated H358 cells.

In TFG-β1-treated A549 cells, SAHA restored H3K79me3 and induced epithelial gene expression but reduced NRP2 and SEMA3C. In combination with DOT1L inhibitors or PFI-1, NRP2 and ZEB1 expression was reduced (Additional file 3 a, b).

These results show that each treatment has benefits and disadvantages to modulate the EMT status or EMT induction. They suggest that SAHA and both combinations of either SAHA/DOT1L or SAHA/PFI-1 inhibitors are potential epigenetic-based therapies to partially reduce or reverse EMT (Table 1).

### H3K79me3 in human normal tissues and cancers

**Fig. 7:**
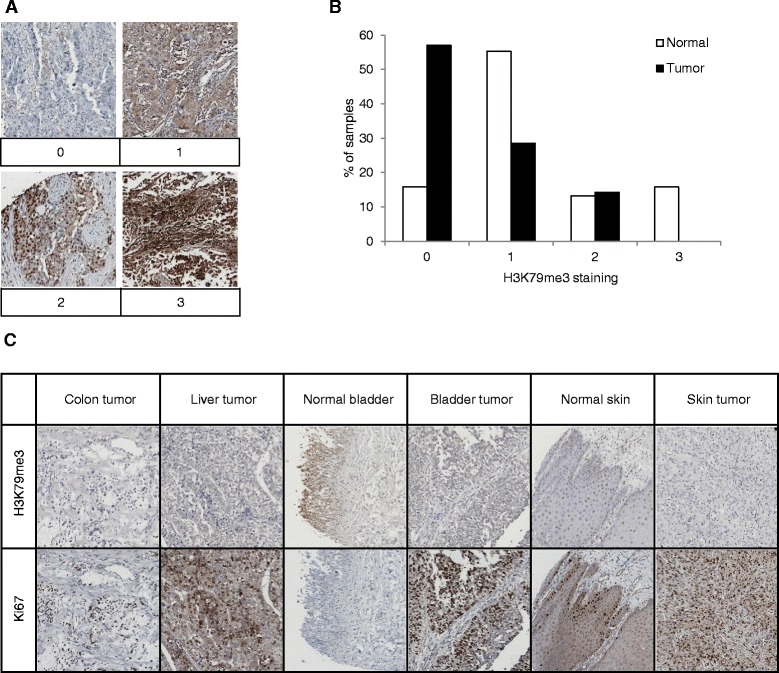
H3K79me3 in human tissues. **a** Examples of H3K79me3 staining intensity (0–3) are given. **b** H3K79me3 staining intensity is shown for normal samples (white, *n* = 38) and tumors (black, *n* = 42) from the multi-tissue TMA BCN962. **c** Examples of H3K79me3 and Ki67 staining from selected normal and tumor samples from the multi-tissue TMA BCN962

Since H3K79me3 was decreased during EMT in cancer cell lines suggesting a relation with aggressiveness, we investigated the presence of H3K79me3 in lung cancers by immunohistochemistry on two commercial TMAs. Because of sample quality, some tumors were excluded, leaving for analysis 17 squamous cell carcinomas, 32 large carcinomas, 48 adenocarcinomas, 3 papillary adenocarcinomas on TMA LC121a, and 11 squamous cell carcinomas, 10 large carcinomas, 12 adenocarcinomas, and 10 small cell carcinomas on TMA MC6163. Scores were obtained by multiplying the percentage of positive (0 to 100%) cells in the tumor compartment by the average level of staining intensity (0 to 3) (Fig. 7a). Histology, tumor grade, and metastatic lymph node did not affect H3K79me3 scores. However, H3K79me3 intensity was statistically higher in large cell carcinomas than in adenocarcinomas in TMA LC121a (median = 2, range 0–3 versus median = 1, range 0–3, respectively, *p =* 0.0149), but the percentage of stained cells was not significantly different suggesting more H3K79me3 in large cell carcinomas. Because of the low number of corresponding samples, this difference was not found on TMA MC6163. In addition, a tendency for less staining was observed for small cell carcinomas. When lung tumors were split into two groups regarding the presence or absence of metastatic lymph nodes, scores were not significantly different.

When the multi-tissue TMA BCN962 was stained for H3K79me3 and Ki67 on two serial slides, both staining did not show the same repartition in normal tissues (*n* = 38) compared to malignant tissues (*n* = 42). H3K79me3 staining was lower in tumors compared to normal tissues (Fig. 7b; *p* = 9.994 × 10^−7^), and in tumors, a negative correlation was found between Ki67 and H3K79me3 (*n* = 42, *r* = −0.52984, *p* = 0.0003). In support of these observations, examples of staining are given for different tumors and normal tissues (Fig. 7c). Interestingly, in the normal skin, strong Ki67-positive cells are localized in the basal cell layer, whereas H3K79me3 staining is faint and extends beyond that zone. These results suggest that H3K79me3 is decreased in tumors compared to that in normal tissues and not associated with cell proliferation.

## Discussion

Epigenetics is involved in EMT to repress epithelial gene expression and to stimulate mesenchymal marker expression and, as a consequence, contributes to cancer invasion, metastasis, immune surveillance escape, and resistance to treatments [6, 7]. In this study using a model of TGF-β1-induced EMT in two NSCLC cancer cell lines, we found a decrease in global H3K79me3 staining with a reduced expression of the corresponding histone methyltransferase DOT1L upon TGF-β1 treatment. Expression of several genes of interest was increased such as PD-L1 involved in the inhibition of immune checkpoints and guidance molecules like SEMA3C and its receptor NRP2. We also found that a co-treatment with DOT1L inhibitors associated with HDAC or a bromodomain inhibitor was efficient to partially reverse modifications in gene expression induced by TGF-β1. In addition, in different human tissues and corresponding tumors, H3K79me3 was generally less in tumors compared to normal tissues, and an inverse correlation was found between H3K79me3 and Ki67, a marker of cell proliferation, suggesting that H3K79me3 is not requested for cell proliferation.

Among genes that showed modified expression upon TGF-β1 treatment, guidance molecules are of particular interest and represent potential new targets in cancers. We showed that both the cell surface receptor NRP2 and SEMA3C (one of NRP2 ligands) were overexpressed during TGF-β1 treatment. NRP2, one of the two members of the neuropilin family, plays an essential role in EMT induction through non-canonical TGF-β1 signaling involving ERK [28]. Neuropilins are pleiotropic cell surface co-receptors for some secreted members of class 3 semaphorins (SEMA3) including SEMA3F and SEMA3C, integrins, and other ligands like VEGF and growth factors such as TGF-β1 [38]. Neuropilins (NRP1 and NRP2) are overexpressed in several cancers, and their expressions correlate with increased invasion and poor prognostic in lung cancer [28, 39]. Recently, it was demonstrated that NRP2 regulates mTOR signaling [40], β-catenin signaling [41], endosome maturation, and EGFR trafficking sustaining cancer development [42]. Therefore, increased NRP2 expression upon TGF-β1 treatment strongly supports a function of NRP2 for TGF-β1 response in EMT induction. Semaphorins were initially described as guidance molecules involved in growth cone migration but were further involved in developmental and pathologic processes including cancer [43–46], and SEMA3C is generally described as a tumor promoting semaphorin [45] Thus, both NRP2 and SEMA3C overexpression might facilitate TGF-β1 signaling and tumorigenesis, and targeting NRP2 and SEMA3C would be useful to reduce EMT.

We also found a significant overexpression of PD-L1 at the membrane of H358 cells during TGF-β1-induced EMT. PD-L1 (also known as B7-H1 or CD274) is one of the ligands of PD-1, an immune checkpoint which prevents T cell activation and limit autoimmunity leading to self-tolerance [47]. We verified that PD-1 was not expressed on H358 cells (data not shown), suggesting absence of autocrine function of PD-L1/PD1 and cell-intrinsic PD-1 pathway as shown in melanoma cells [48]. PD-L1 is overexpressed in cancer and is involved in tumor immune escape, leading to cancer development and metastasis [30, 49, 50]. It was associated with poor clinical outcomes in several types of cancer, including NSCLC [29, 51–53]. By an integrated analysis of three independent large datasets, PD-L1 was associated with lung adenocarcinomas displaying a “mesenchymal” phenotype [30]. Indeed, a molecular link was found between PD-L1 and EMT where PD-L1 is negatively regulated by miR-200, and this inhibition is relieved by ZEB1, an EMT activator induced by TGF-β1 [54]. PD-L1 upregulation would be a consequence of EMT induction. Therefore, compounds that partially reverse EMT would target PD-L1 as well and reduce tumor progression and metastasis by restoring the immune response. In fact, pembrolizumab and nivolumab targeting PD-1 were recently approved for NSCLC to prevent PD-L1 binding [55].

Histone methylation is a well-balanced mechanism guided by histone methyltransferases and demethylases. Disruptor of telomeric silencing 1-like (DOT1L) is a histone methyltransferase for H3K79, and its recruitment is ubiquitously coupled with transcription. Strong similarities were noticed between H3K79 and H3K4 methylation patterns, and H3K79me3, although present at very low levels, peaks just behind the transcriptional start site and gradually decreases on the gene body [56, 57]. However, the role of DOT1L in gene expression is still controversial, but reports of H3K79me3 association with gene repression might be linked to technical issues raised by the antibody quality and absence of SDS during chromatin preparation [56]. DOT1L is also involved in chromosome integrity and heterochromatin formation. DOT1L is well-known to be associated with pathological functions where interactors recruit DOT1L to specific gene regions, like in MLL-rearranged leukemias which present abnormal H3K79 methylation pattern at HOX loci [58–61]. In breast cancers, DOT1L is associated with poorer survival and aggressiveness, and DOT1L can cooperate with c-MYC and histone acetyltransferases to activate EMT and enhance cancer stem cell-like properties [62]. In lung adenocarcinomas, DOT1L has been recently described to be mutated in 3% of tumors, suggesting abnormal H3K79me in a subset of samples [17]. In contrast to breast cancers, we found a decrease of global H3K79me3 in TFG-β1-induced EMT in NSCLC cells. We suggest that H3K79me3 decrease is a mark of TGF-β1 treatment and not a general mark of EMT since additional NSCLC cell lines with a mesenchymal phenotype like H661 and H460 show higher H3K79me3 than the more epithelial H358 cells (Additional file 4). In addition, H3K79me3 level does not correlate with DOT1L expression (Additional file 4). Therefore, DOT1L activity and association with specific partners need to be considered. Also, RE-IIBP expression and activity should be studied since it was recently identified as a histone methyltransferase for H3K79 [63]. In addition, H3K79me3 demethylation could be involved in H3K79me3 decrease, but no specific demethylase has been described to our knowledge yet. Cross-talks with other histone marks should be considered as well. Indeed, H2B ubiquitination is a prerequisite for DOT1L enzymatic activity [35]. Although we did not find gross difference of H2BK120ub level in H358 and A549 cells after TGF-β1 treatment, H2BK120me1 and HBK120ac were decreased suggesting more complex cross-talks between histone marks.

In lung tumors, H3K79me3 intensity was statistically higher in large cell carcinomas than in adenocarcinomas. Would this difference be related to poor differentiation? The answer cannot be given because of lack of information with commercial TMAs about the status of BRG1, MYC, and EGFR. Of note, loss of function of BRG1 in about 12% of lung adenocarcinomas was shown to be associated with poor differentiation with EMT features and poor survival [64, 65]. Considering that up to one third or more of large cell carcinomas have alteration of BRG1 or other SWI/SNF subunits, it seems reasonable to consider that large cell carcinomas show poor differentiation. However, to associate DOT1L with poor differentiation would be in contradiction with the fact that DOT1L reduced cell reprogramming from a somatic differentiated state to an undifferentiated pluripotent state in several models [61]. In addition, we found H3K79me3 decrease in EMT known to generate gain of stem cell properties in models including immortalized mammary epithelial cells [66]. Therefore, better characterization of these large cell carcinomas is requested since, before the recent 2015 WHO classification [67], they were a heterogeneous group of tumors that makes interpretation difficult with the samples we analyzed.

The presence or absence of metastatic lymph nodes did not affect H3K79me3 scores in primary tumors. Interestingly, in a panel of human normal tissues and cancers, H3K79me3 level seems to be less present in tumors in comparison to normal tissues and not associated with cell proliferation. However, in both H358 and A549 cell lines, cell proliferation is not the cause of H3K79me3 decrease as it was not affected by TGF-β1 for 48 h.

We tried epigenetic treatments to modulate EMT. DOT1L inhibitors did not induce EMT as might be expected if H3K79me3 decrease was one of the causes of EMT. They were rather not effective on EMT target genes. Of interest, DOT1L was recently shown to methylate non-histone proteins like the androgen receptor through recruitment of the PRNCR1 long non-coding RNA for subsequent activation of this receptor in prostate cancer after looping between enhancer and promoter sequences [68]. Therefore, complex responses are expected with DOT1L inhibitors. Targeting selective histone methyltransferase is a promising approach, and DOT1L inhibitors are in clinical trials for leukemias with MLL rearrangements [69].

The best combination to partially reverse TGF-β1-induced EMT to a more epithelial phenotype was SAHA (a HDAC inhibitor) associated with a bromodomain inhibitor. With this treatment, NRP2, SEMA3C, and PD-L1 expression were reduced. For other classical EMT genes, the response was gene-specific. More experiments would be necessary with dose response for each compound alone or in combination, associated with functional tests for cell migration and invasion. In addition, this first screening would be improved by use of a 3D cell culture model. Several studies highlight the benefits of epigenetic-based therapeutic strategy in mouse models. Combining the bromodomain inhibitor JQ1 with the histone deacetylase inhibitor SAHA in pancreatic cancer inhibits both MYC activity and inflammatory signals as well as in an established adenocarcinoma lung cancer model with KRas^G12D^ mutation and p53 loss [36]. In a neuroblastoma mouse model, JQ1 and panabinostat (another HDAC inhibitor) in combination, synergistically reduced N-MYC protein and tumor progression [37]. As MYC is often overexpressed in tumors including lung cancers and directly binds PD-L1 promoter [70], these co-treatments are expected to reduce PD-L1 expression. From a mechanistic point, bromodomains might be efficient by targeting BRD4 binding to acetylated H4K5 at proximity to superenhancers at highly transcribed genes. Di-methylation of H3K79 mediated by DOT1L is involved in this process, as described in MLL leukemia, suggesting an unrecognized functional interplay between BRD4 and DOT1L [60].

## Conclusion

In summary, we described epigenetic changes that decrease global methylation of H3K79 during TGF-β1-induced EMT, and we suggest a potential therapy with HDAC inhibitors associated to a bromodomain inhibitor to partially reverse EMT. However, clinical applications need careful characterization of tumors, and caution must be exerted with these treatments not to induce MET (mesenchymal-to-epithelial transition), particularly in cases in which tumor cells have already disseminated [71, 72].

## Additional files

Additional file 1: List of antibodies. (PDF 235 kb)

Additional file 2: RT-qPCR primer list and sequences. (PDF 199 kb)

Additional file 3: Partial reversion of TGF-β1-induced EMT by epigenetic inhibitors inA549 cells. (PDF 445 kb)

Additional file 4: H3K79me3 and DOT1L level. (PDF 274 kb)

## Abbreviations

EMT: Epithelial-to-mesenchymal transition
miR: micro RNA
NRP2: Neuropilin-2
NSCLC: Non-small lung cancer cell
PD-L1: Programmed death-ligand 1
PTM: Post-translational modification
