# Supplementary material for: Tri-methylation of H3K79 is decreased in TGF-β1-induced epithelial-to-mesenchymal transition in lung cancer
Source: Clin Epigenetics. 2017 Aug 8;9:80. doi: 10.1186/s13148-017-0380-0 (PMC5549304; doi:10.1186/s13148-017-0380-0)
Supplement: Supplementary file 1 — List of antibodies. (PDF 235 kb) [file 13148_2017_380_MOESM1_ESM.pdf]

Additional file 1: list of antibodies

Protein	MW (kDa)	Compagny	Reference	Western Blot	Flow cytometry	ICC
β-actin	42	Sigma-Aldrich	A5441	1:40 000		
BB515 IgG1, Isotype Control		BD Biosciences	564416		5 µl for 10^6cells	
DOT1L	185	Novus Biologicals	NB100-40845	1:500		
E-cadherin	120	Bdbioscience	610181	1:5 000		
H2AK119ub1	26	Cell Signaling	8240s	1:1 000		
H2BK120ac	17	Active Motif	39119	1:1 000		
H2BK120me1	17	given by Ryuji Hamamoto		1:500		
H2BK120ub1	26	Active Motif	36623	1:1 000		
H3 Total	17	Active Motif	39163	1:10 000		
H3K27Ac	17	Active Motif	39135	1:1 000		
H3K27me1	17	Active Motif	61015	1:1 000		
H3K27me3	17	Active Motif	39156	1:1 000		
H3K36me1	17	Active Motif	61351	1:1 000		
H3K36me2	17	Active Motif	39255	1:2 500		
H3K36me3	17	Active Motif	61101	1:2 000		
H3K4me1	17	Active Motif	39297	1:1 000		
H3K4me2	17	Active Motif	39141	1:1 000		
H3K4me3	17	Active Motif	39159	1:1 000		
H3K79me1	17	Active Motif	39145	1:1 000		
H3K79me2	17	Active Motif	39143	1:1 000		
H3K79me3	17	Diagenode	pAb-068-050	1:500		1:200
H3K9Ac	17	Active Motif	39137	1:1 000		
H3K9me1	17	Active Motif	39887	1:1 000		
H3K9me2	17	Active Motif	39375	1:5 000		
H3K9me3	17	Active Motif	39161	1:1 000		
N-cadherin	100	Abcam	1224	1:5 000		
PD-L1	50	Cell Signaling	13684	1:500		1:50
PD-L1	50	Cell Signaling	564554		5 µl for 10^6cells	
Vimentin	57	Cell Signaling	R28	1:1 000		
Peroxidase-conjugated AffiniPure Goat anti-Rabbit IgG		Jackson immunoresearch	111-035-003	1:20 000		
Peroxidase-conjugated AffiniPure Goat anti-Mouse IgG		Jackson immunoresearch	115-035-003	1:20 000		
Chicken anti-Rabbit IgG (H+L) Secondary Antibody, Alexa Fluor 488		ThermoFisher Scientific	A-21441			1:200
