# Supplementary material for: Tri-methylation of H3K79 is decreased in TGF-β1-induced epithelial-to-mesenchymal transition in lung cancer
Source: Clin Epigenetics. 2017 Aug 8;9:80. doi: 10.1186/s13148-017-0380-0 (PMC5549304; doi:10.1186/s13148-017-0380-0)
Supplement: Supplementary file 2 — RT-qPCR primer list and sequences. (PDF 199 kb) [file 13148_2017_380_MOESM2_ESM.pdf]

Additional file 2: RT-qPCR primer list and sequences

Primer name	Forward 5' to 3'	Reverse 5' to 3'
DOT1L	GACCCCTCGTCCACACTTG	CCAGCGTAGGAGAAGCCTG
E-cadherin	CGGGAATGCAGTTGAGGATC	AGGATGGTGTAAGCGATGGC
EPCAM	CGCAGCTCAGGAAGAATGTG	TGAAGTACACTGGCATTGACGA
ESRP1	TCCTGCTGTTCTGGAAAGTCG	TCCGGTCTAACTAGCACTTCGTG
Gapdh	AGGTGAAGGTCGGAGTCAACG	CGTTCTCAGCCTTGACGGTG
Neuropilin 2	GGATGGCATTCCACATGTTG	ACCAGGTAGTAACGCGCAGAG
PD-1	GACAGCGGCACCTACCTCTGTG	GACCCAGACTAGCAGCACCAGG
PD-L1	CAATGTGACCAGCACACTGAGAA	GGCATAATAAGATGGCTCCCAGAA
Rab25	AATGTTCGCTGAAAACAATGGAC	CTCAAAGGCTAGCTCAACATTGG
SEMA3C	ATCGAGTGAACGCTGCTGATG	GCTCGCCACTGACAGAGTTGTT
SEMA3F	ACACCATGGACCCAGACTCAG	GAAGATGTAATCCTGGCGTG
ST14	GGGACACACCCAGTATGGAGG	GAGGTTCTCGCAGGTGGTCTG
Vimentin	TTTGAAGAAACTCCACGAAGAGGA	CCACATCGATTTGGACATGCT
Zeb1	AGCAGTGAAAGAGAAGGGAATGC	GGTCCTCTTCAGGTGCCTCAG
