# Supplementary material for: Tri-methylation of H3K79 is decreased in TGF-β1-induced epithelial-to-mesenchymal transition in lung cancer
Source: Clin Epigenetics. 2017 Aug 8;9:80. doi: 10.1186/s13148-017-0380-0 (PMC5549304; doi:10.1186/s13148-017-0380-0)
Supplement: Supplementary file 3 — Partial reversion of TGF-β1-induced EMT by epigenetic inhibitors inA549 cells. (PDF 445 kb) [file 13148_2017_380_MOESM3_ESM.pdf]

Additional file 3: Partial reversion of TGF-β1-induced EMT by epigenetic inhibitors inA549 cells. (A) Cells were treated simultaneously with TGF-β (10 ng/ml), EPZ5676 (1 μM), SGC0946 (5 μM) or PFI-1 (5 μM) for 48 hours, and with SAHA (5 μM) for the last 12 hours. Proteins were analyzed by immunoblotting of total cell lysates. Actin was used as a loading control. The apparent molecular weights (kDa) are indicated on the right of the panel. (B) mRNA expression was measured by RT-qPCR and normalized to the GAPDH mRNA for control cells (white bars) and TGF-β1 treated cells (black bars). The graph corresponds to the mean ± SD of two independent experiments with qPCR in duplicate**.** *$p < 0.5$, ** $p < 0.01$, *** $p < 0.001$, **** $p < 0.0001$ by Two-way ANOVA test.

A

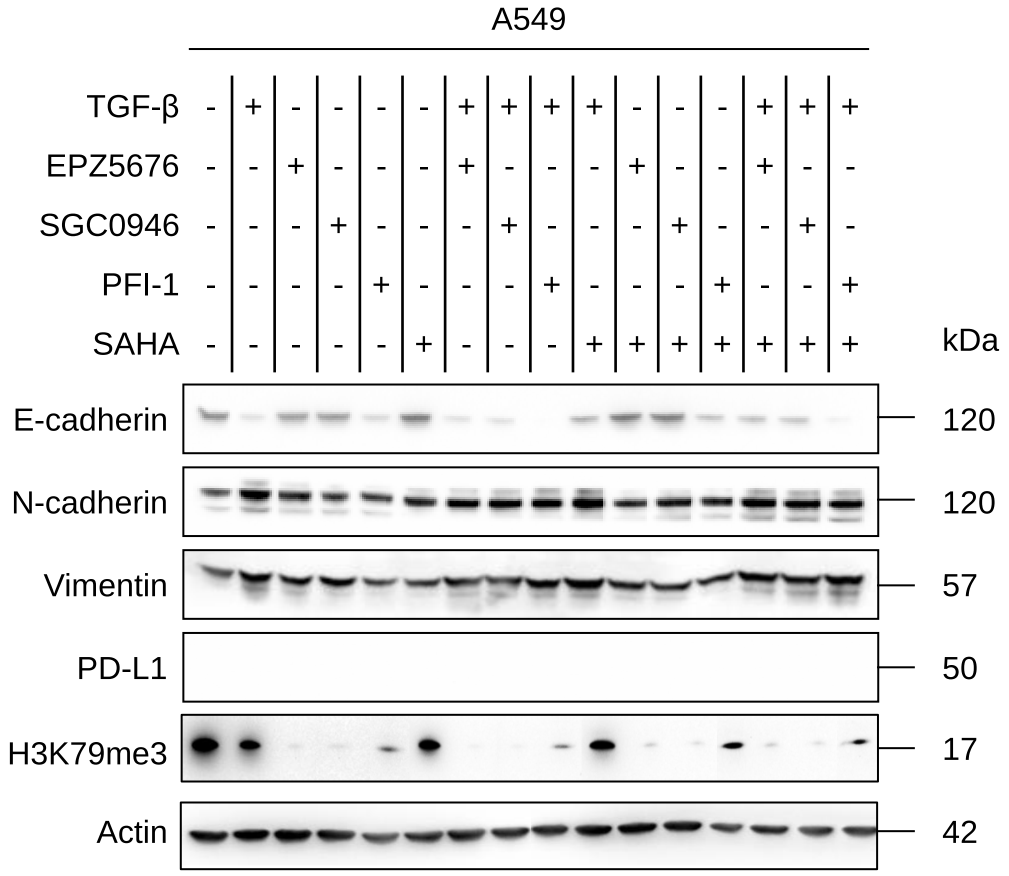

B

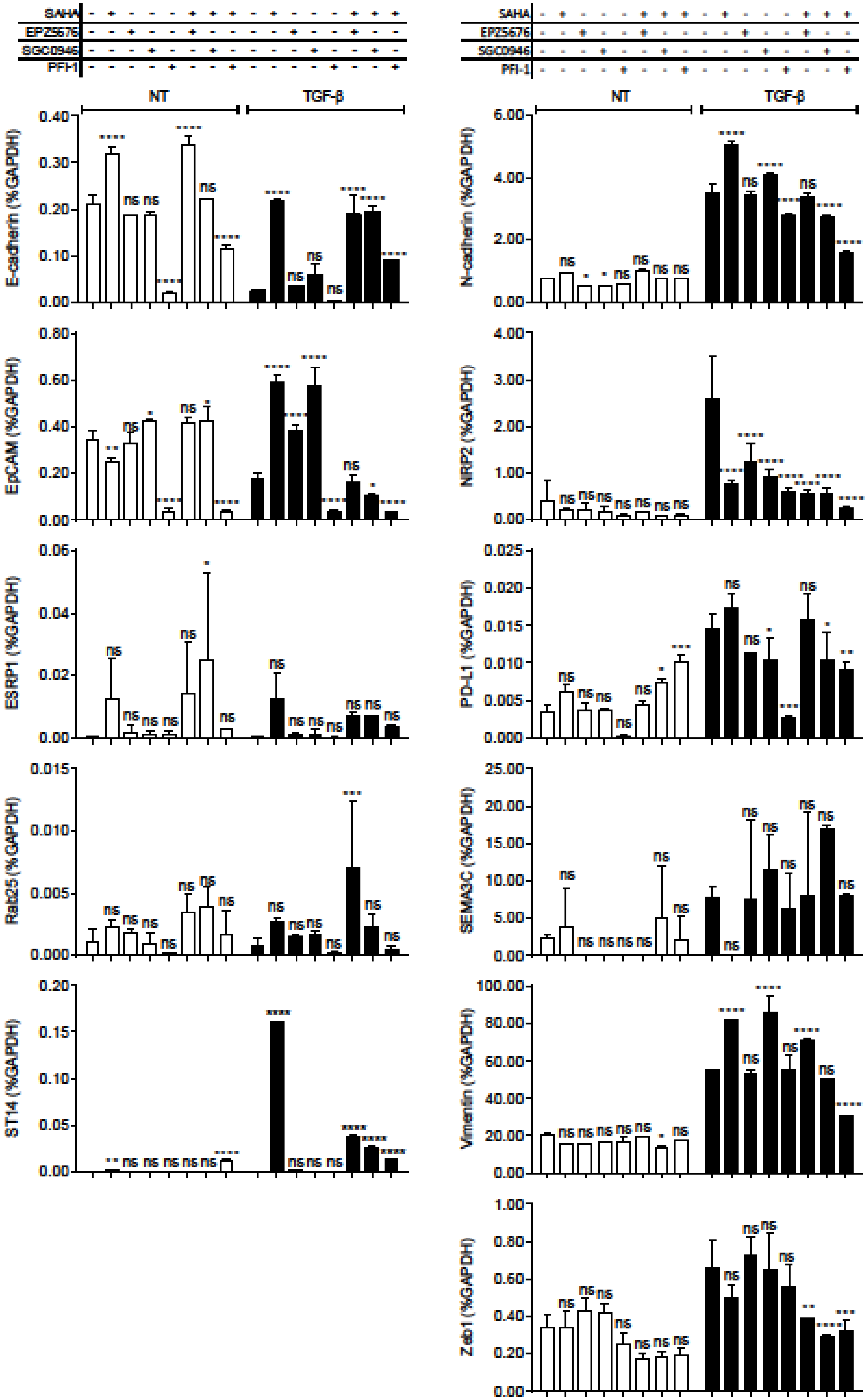

SAHA
EPZ5676
SGC0946
PFI-1
NT
TGF-β
E-cadherin (%GAPDH)
EpCAM (%GAPDH)
ESRP1 (%GAPDH)
Rab25 (%GAPDH)
ST14 (%GAPDH)
N-cadherin (%GAPDH)
NRP2 (%GAPDH)
PD-L1 (%GAPDH)
SEMA3C (%GAPDH)
Vimentin (%GAPDH)
Zeb1 (%GAPDH)
