# Supplementary material for: Tri-methylation of H3K79 is decreased in TGF-β1-induced epithelial-to-mesenchymal transition in lung cancer
Source: Clin Epigenetics. 2017 Aug 8;9:80. doi: 10.1186/s13148-017-0380-0 (PMC5549304; doi:10.1186/s13148-017-0380-0)

Additional file 4: H3K79me3 and DOT1L level. A549 and H358 cells were treated with TGF-β (10 ng/ml) for 48 hours. Proteins were analyzed by immunoblotting of total cell lysates. Actin was used as a loading control. The apparent molecular weights (kDa) are indicated on the right of the panel.

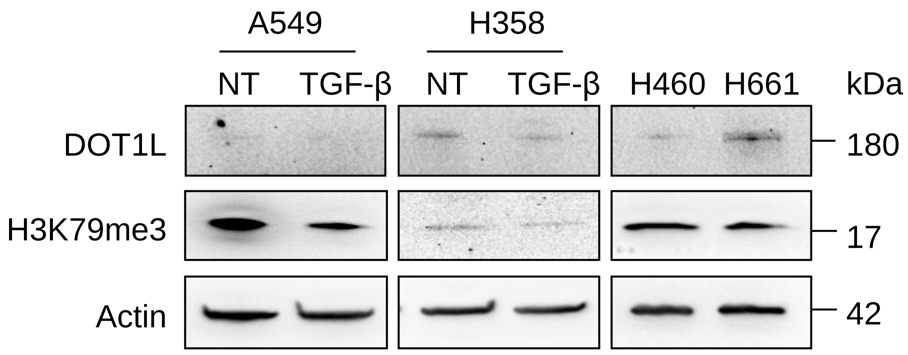

Supplement: Supplementary file 4 — H3K79me3 and DOT1L level. (PDF 274 kb) [file 13148_2017_380_MOESM4_ESM.pdf]
